# Testing the Traditional Chinese Medicine Consultation Model for Adherence in Complementary and Alternative Medicine

**DOI:** 10.1155/2020/8897628

**Published:** 2020-12-30

**Authors:** Amally Ding, Jignesh P. Patel, Vivian Auyeung

**Affiliations:** ^1^Institute of Pharmaceutical Science, King's College London, London SE1 9NH, UK; ^2^Department of Haematological Medicine, King's College Hospital, London SE5 9RS, UK

## Abstract

The Traditional Chinese Medicine (TCM) Consultation Model for Adherence conceptualises the consultation process specific to patient adherence. It can be used to improve patient persistence with treatment by TCM practitioners and possibly other health professionals. The aim of this research was to determine the applicability of the TCM Consultation Model for Adherence in the wider complementary and alternative medicine (CAM) setting. A survey containing validated questionnaires and items developed specifically to test the model was administered online in the United Kingdom. SPSS 25 was used to perform Spearman's correlations and Mann–Whitney *U* tests on the data. In total, 101 patients completed the survey. The results showed that patients having a therapeutic relationship and trusting in their practitioner was associated with overall adherence to CAM, while patients feeling supported was associated with all types of adherence to CAM. Specific behaviours of the TCM Consultation Model for Adherence that were positively correlated with adherence to CAM were identified. They could potentially be used by CAM practitioners to improve their patients' adherence with treatment.

## 1. Introduction

Adherence is the similarity between the actions of a patient and the advice offered by their health professional, which was agreed upon together for implementation [1]. Nonadherence is an issue in healthcare that affects all types of medicine prescribed for chronic conditions [1, 2]. The adherence rate is reported to be 50% for both conventional medicine and also herbs or remedies in complementary and alternative medicine (CAM) [1, 2]. The consequence of inadequate adherence is ultimately poorer patient health [1].

There is a plethora of models for understanding adherence and for the consultation, yet only one that describes how factors in the consultation affect patient adherence with treatment: the TCM Consultation Model for Adherence [3]. It is grounded in observations and qualitative interview data [3]. At the core of the model is patients feeling cared for [3]. Patients also needed to feel comfortable and valued as individuals, encompassing feeling understood, as well as known and supported in the management of their health [3]. With trust, a therapeutic relationship could then be established between the patient and TCM practitioner to enhance adherence with treatment [3]. Although the model emerged from within one CAM setting, it could prove useful for other CAM practitioners as a guide to increase patient adherence with the treatments they prescribe [3].

The aim of this study was to determine whether the TCM Consultation Model for Adherence can be applied across CAM. The following hypotheses were tested as part of the study. There is a positive association between

1. Patients feeling cared for and overall adherence
2. Patients feeling comfortable and overall adherence
3. Patients feeling valued as individuals and overall adherence
4. Patients feeling understood and overall adherence
5. Patients feeling known and overall adherence
6. Patients feeling supported in the management of their health and overall adherence
7. Patients trusting in their practitioner and overall adherence
8. Patients having a therapeutic relationship with their practitioner and overall adherence

## 2. Materials and Methods

### 2.1. Design

A self-report survey was developed to encompass questions on all of the factors from the TCM Consultation Model for Adherence, extent of adherence to treatment, and use of CAM and sociodemographics. The most relevant validated questionnaires in the CAM literature were adopted and items created where none were suitable. Aspects of the TCM Consultation Model for Adherence were mapped to all items of validated questionnaires at face value by the primary researcher. Items developed specifically for the study tested novel aspects of the TCM Consultation Model for Adherence and related concepts in the literature that were previously untested. The survey was piloted on three potential participants. Their feedback was incorporated where possible, such as improving wording unless part of a standardised questionnaire.

### 2.2. Measures

The four standardised instruments used were the 39‐item checklist of CAM modalities [4], the adherence measure developed by Bishop et al. [5], the Consultation and Relational Empathy (CARE) Measure [6], and Perception of Therapist subscale from the Treatment Appraisal Questionnaire [5].

The 39‐item checklist of CAM modalities captures all the CAM treatments primarily tried by participants in the United Kingdom [4]. The checklist has been used in a few studies [5, 7, 8], with one reporting all alphas above 0.85 [8]. Cronbach's alpha was 0.86 in this study.

The adherence measure developed by Bishop et al. [5] consists of three items where patients rate the extent of advice given by their CAM practitioner followed on a seven point scale from not at all (1) to completely (7). The three items cover three types of adherence. Thus, one item was on appointments, another on lifestyle advice, and the last on remedies. The measure was deemed to be more meaningful for use in CAM than other validated measures, which focus on medication. However, it does not contain an overall adherence item. Thus, the median score of the three items has been taken as the overall adherence score. Cronbach's alphas were not reported in the two studies [5, 9] that have used the adherence measure developed by Bishop et al. [5].

The CARE Measure is a 10 item questionnaire asking patients to rate the interpersonal aspects of their healthcare provider on a scale from poor to excellent [6]. It was developed to assess general practitioners but is used in CAM, as one of the first few papers supporting CARE Measure use was in acupuncture [10]. Cronbach's alpha was reported to be 0.93 in the study first validating its use [6] and 0.92 in this study.

The Perception of Therapist subscale from the Treatment Appraisal Questionnaire, which consists of 10 items from the 20, asks patients to rate the interpersonal aspects of their CAM practitioner on a seven-point Likert scale [5]. It has been used previously [9, 11], and Cronbach's alpha was 0.91 for the pilot study [5], while it was 0.88 in this study.

### 2.3. Recruitment

Patients aged 18 years or above receiving CAM treatment for their own condition were recruited from August 2018 to January 2019 in the United Kingdom. Patients were excluded if they were under 18 years of age or seeking treatment on behalf of another. Patients with either acute or chronic conditions were included. Participants self-identified or were referred by their practitioner. As the cross-sectional survey was administered on SurveyMonkey, the link was sent along with information about the study through organisation distribution lists and posted on social media or other websites. The most popular or recognised association from each type of CAM listed under the Research Council for Complementary Medicine was contacted in addition to other organisations that were further recommended by these associations. General public websites were also approached, and the study was successfully advertised through a university email circular. Each participant who completed the anonymous survey was offered entry into a £50 store voucher prize draw.

### 2.4. Analysis

The data were analysed in IBM SPSS Statistics for Windows, Version 25.0. Responses were excluded if an entire measure or more was incomplete and when the participant was ineligible. Missing values were excluded from analysis or imputed according to the scoring guideline of the measure. Specifically, when ‘Does not apply' was selected by a respondent for one or two items on the CARE Measure, it was replaced with the average score of the remaining items in the measure for the individual [12]. If there were more than two items with ‘Does not apply' selected by an individual, the participant's response was removed from analysis [12].

Where answers were in the free text format, they were coded into categories. Occupations were coded according to the sections of the International Standard Industrial Classification of All Economic Activities Revision 4 [13]. Health problems were coded using the chapters of the International Statistical Classification of Diseases and Related Health Problems 10th Revision [14]. Additional categories were created where none were suitable, such as ‘Multiple' when more than one health problem or occupation was listed by an individual.

Aside from reporting descriptive statistics for the data, Spearman's correlation tests were applied to items assessing the therapist, consultation, and clinic experience against levels of adherence to determine if there were any associations. The strengths of the resultant correlations were then interpreted according to the reference values commonly accepted by the research field [15]. In psychology, correlation coefficients <0.4 are weak, 0.4–0.7 are moderate, and >0.7 are strong [15]. Mann–Whitney *U* tests were applied to therapist traits against the degree of adherence to determine if there were any associations with the binary data. Significance levels were reported along with the test values. To protect against familywise error from multiple hypothesis testing, the convention of *p* < 0.01 was selected as a compromise between a strict Bonferroni correction and *p* < 0.05 in the related literature [5]. To protect against type II errors, power analysis calculations were performed to confirm the study had over 80% power to detect the average statistically significant correlation for overall adherence. The tests selected were the most suitable for the sample in terms of size and nonnormal distribution.

### 2.5. Ethics

Prior to study commencement, ethical approval was granted by the King's College London Biomedical Sciences, Medicine, Dentistry, and Natural and Mathematical Sciences Research Ethics Panel (LRS-17/18–7527).

## 3. Results

### 3.1. Sociodemographics

During the 6 months it was open, 101 eligible participants completed the survey. The median age of the sample was 49 years (range: 21–74). The vast majority of participants were female (90.1%) and white (86.1%). Most of the participants had undertaken tertiary education (94.1%) with 59.4% having, at least, a bachelor's degree. Participants predominantly worked in the human health and social work activities industry (42.6%). The highest proportion was comprised of CAM therapists (28.7%). Participants' annual income was generally under £30000. The sociodemographic details can be found in Table 1.

The main problem patients sought treatment for were diseases of the musculoskeletal system and connective tissue (30.7%), as seen in Table 2. The median duration for which patients have seen their current therapist was 3 years (range: 1 day–20 years).

Almost half of the sample were receiving massage (47.5%) from their therapist. Reflexology (20.8%) was the next most popular treatment, followed by acupuncture (19.8%). Participants usually received more than one treatment modality at a time from their therapist. More than half of the sample had tried yoga (51.5%), reflexology (51.5%), massage (51.5%), and acupuncture (50.5%) in the past. This is shown in Table 3. A list of all the CAM treatment modalities used and tried by participants is in Appendix A.

### 3.2. Adherence

Participants indicated they were very willing to make sacrifices to use CAM (*M* = 6, IQR = 5–7). Close to half of the sample had been advised to use a remedy (48.5%), while most had been advised to make changes to lifestyle (86.1%) and follow-up appointments (91.1%). Overall adherence was high (*M* = 6, IQR = 5–7). Specifically, remedy adherence was high (*M* = 6, IQR = 5–7). Lifestyle advice had the lowest rating of the three adherence types (*M* = 5, IQR = 5–6), whilst appointment adherence was complete in more than half of the respondents (*M* = 7, IQR = 6–7).

### 3.3. The Consultation and Therapist Experience

Patients generally rated their therapist extremely well. Aside from four items, the highest possible rating was given for all other items in the survey. Ratings of 5 or 6 were given for the four items: “My therapist is an expert in my treatment,” “My therapist knows how to treat my health problem,” “My therapist shares the same values as me,” and “My therapist offers advice beyond the immediate health problem”.

#### 3.3.1. The CARE Measure

Participants reported that their therapist was excellent at showing empathy. Every item on the CARE Measure in Table 4 typically received an excellent rating.

#### 3.3.2. The Perception of Therapist Subscale

Patients had positive perceptions of their therapist. Being an expert and knowing how to treat the patient's health problem were the two items where the majority did not completely agree on the Perception of Therapist subscale in Table 5. Patients tended to mostly agree with these two statements.

#### 3.3.3. Additional Items

Additional items evaluating the therapist and clinic were usually highly rated. They can be found in Table 6. All statements except “My therapist shares the same values as me” and “My therapist offers advice beyond the immediate health problem” had the majority in complete agreement.

Almost all patients thought their therapist was a good person and calm, confident, and professional (>90%). Aside from being considered a wise healer and sharing the same gender, other roles and traits were not present in more than half of the patients' therapists. This can be seen in Table 7.

### 3.4. Highlights

- Most factors of the TCM Consultation Model for Adherence did not appear to be associated with adherence to CAM, although some of the behaviours that exemplify factors were found to be positively correlated.
- Patients feeling supported in the management of their health was the only factor that consistently showed weak to moderate correlation across all adherence types.
- Overall, patients trusting in their practitioner and having a therapeutic relationship were associated with adherence. However, their statistical significances varied across the individual types of adherence.
- A summary of the aspects of the TCM Consultation Model for Adherence found to be statistically significant for each adherence type is in Table 8.

### 3.5. Hypotheses Testing

From investigating the relationship between aspects of the TCM Consultation Model for Adherence and adherence to CAM, it was found that there was

1. No association between patients feeling cared for and overall adherence (*r*_s_ = 0.237, *p*=0.017)
2. No association between patients feeling comfortable and overall adherence (*r*_s_ = 0.108, *p* < 0.281)
3. No association between patients feeling valued as individuals and overall adherence (*r*_s_ = 0.174, *p* < 0.082)
4. No association between patients feeling understood and overall adherence (*r*_s_ = 0.191, *p*=0.056)
5. No association between patients feeling known and overall adherence (*r*_s_ = 0.174, *p*=0.082)
6. A positive association between patients feeling supported in the management of their health and overall adherence (*r*_s_ = 0.357, *p* < 0.001)
7. A positive association between patients trusting in their practitioner and overall adherence (*r*_s_ = 0.353, *p* < 0.001)
8. A positive association between patients having a therapeutic relationship with their practitioner and overall adherence (*r*_s_ = 0.309, *p*=0.002)

**Figure 1:**
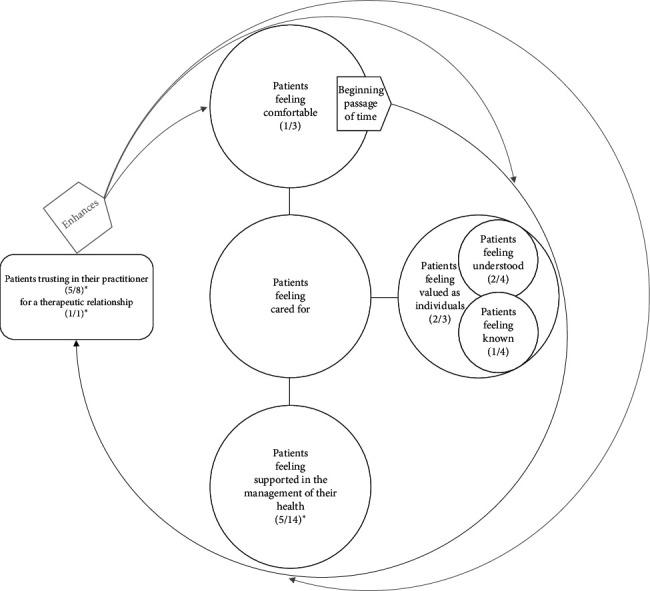
The TCM Consultation Model with statistically significant aspects highlighted for overall adherence in CAM. Note: inside the brackets, the numerator is the number of statistically significant associations with overall adherence, while the denominator is the number of items that have been tested under each factor. ^*∗*^indicates a statistically significant association between a factor and overall adherence at *p* < 0.01.

Details of the associations between aspects of the TCM Consultation Model for Adherence and the types of adherence are in Appendix B.  Table 9 lists all the associations.  Figure 1 summarises the findings.

## 4. Discussion

This study aimed to test the applicability of the TCM Consultation Model for Adherence in the broader CAM setting.

Unexpectedly, the core factor of the TCM Consultation Model for Adherence was not found to be associated with adherence in CAM. Most factors of the TCM Consultation Model for Adherence did not show statistical significance in relation to CAM adherence. Patients feeling supported in the management of their health was the only factor consistently found to be correlated with all types of adherence, while trust and the therapeutic relationship were for overall and certain types of adherence.

The therapeutic relationship has been found to enhance patient adherence with CAM in the literature. Qualitative and quantitative studies show a direct impact [16, 17]. This is consistent with overall adherence in the current study, but not between the types. Although there may, in fact, be no association between the therapeutic relationship and lifestyle advice or appointment adherence, the cause is most likely due to the low *p* value set at 0.01, protecting against familywise error. A larger sample would offer clarity on the matter, as types of adherence have not previously been examined for association with the therapeutic relationship.

In contrast, trust has been tested for association with types of adherence [5]. It was found positively correlated with adherence to appointments [5]. This is consistent with the findings from the current study. However, trust was also associated with overall and, specifically, remedy adherence. No other study has repeated the measure, which makes it difficult to explain the inconsistency. Yet qualitatively, there is more evidence reporting the contribution of trust directly on CAM adherence as a whole [16, 18].

From the findings of this study, it can be seen that the factor that was consistently correlated with adherence across its types was patients feeling supported in the management of their health. Helping as much as possible but knowing limits, were representative behaviours. Although the survey item indicative of support has been used previously, it was reported as part of the Perception of Therapist subscale. Thus, comparisons could not be made to corroborate the finding. Despite the lack of corroborating literature, there are a number of studies illustrating the importance of support in the consultation process [19] and treatment outcomes [20]. This is akin to the studies on the therapeutic relationship and trust, which is also often described to be a part of the therapeutic relationship [20, 21]. They show that aspect is important without touching upon adherence. However, the issue here appears to be complicated by the lack of a definition for ‘support'. For example, the therapeutic relationship has been described as an aspect of support [20]. The Perceived Provider Support Scale should presumably be used to measure patients feeling supported in the management of their health in this study, but its items do not correspond neatly to the subfactors of the TCM Consultation Model for Adherence [20]. “My therapist cares about me” and “I feel cared for during treatment” would belong to the category of patients feeling cared for, not under patients feeling supported in the management of their health according the TCM Consultation Model for Adherence [20]. There was overlap of items with other scales too, so the Perceived Provider Support Scale was not used in this study to reduce redundancy and patient burden.

A related concept worth mentioning is empowerment which is a broader term for enablement [20]. Empowerment mediates the effect of practitioner support [20], while enablement has been found to mediate empathy albeit not definitively [10, 22]. However, the outcome of interest in these studies was not adherence, and there was overlap in definition of support between factors of the TCM Consultation Model for Adherence [10, 20, 22]. Thus, enablement and empowerment were not tested in this study. The behaviour for empathy that was found to be associated with adherence in this study was touching appropriately. The exception was for remedy adherence. The explanation could be due to the therapy type in that most respondents were receiving touch-based therapies, so appropriate physical contact was important. The use of remedies was not seen to require physical contact, evidenced in another study where difficulty traveling to appointments predicted adherence to remedies [5].

There are known differences in patient preference for the type of support between CAM. For example, emotional support is not desired in osteopathy [17] but in TCM [3, 23]. The variation could explain why only a few behaviours showed statistically significant association between support and adherence in this study. The way patients' therapists talk about health problems made sense was consistently associated with adherence across its types. Yet, explaining things clearly was statistically significant for remedy adherence, but in contrast to other types of adherence, where providing explanations of treatment that make sense specifically was statistically significant. Less emphasis on explanations of remedies may be because of familiarity with medicine being prescribed by doctors, although there are different attitudes towards the two types of medicine [3].

Tailoring explanations of treatment is not the only area where CAM practitioners can focus their efforts to enhance adherence. Individualisation of treatment itself is as well. It is known to affect treatment outcomes. However, due to the *p* value set at <0.01 in this study, it was found to be associated to all types of adherence except appointments (*p*=0.01). Nevertheless, CAM practitioners checking treatment was consistently correlated with patient adherence, including all types.

The idea of adapting to the needs of the patient is prevalent in CAM [24]. This could be why one presenting manner was not found to be associated with adherence over another. The exception is positivity, which has been quantitatively found to influence treatment outcomes through enablement [10].

Specific behaviours, rather than overarching feelings, appear to drive adherence in CAM. It can be seen with opening patients up about themselves, rather than patients feeling comfortable, being associated with adherence. If the *p* value was set at ≤ 0.01, then lifestyle advice would be included for all types of adherence to be found correlated with the behaviour. However, there does appear to be nuances between the types of adherence not attributed to chance [18]. Differences in adherence types can vary by CAM treatment modality [18]. The heterogeneity of the CAM treatment modalities may have diluted the importance of the factors found motivating adherence in TCM.

Nonetheless, there are behaviours from the TCM Consultation Model for Adherence that CAM practitioners can enact to improve patient adherence with therapy. Besides opening patients up about themselves, CAM practitioners can tailor treatments to the individual patient, check these treatments, take patients' concerns seriously, touch them appropriately, have a positive manner, explain in a way that makes sense to the patient, and help as much as possible, but within limits.

The main limitation of the study is related to the sample. A larger sample may have helped clarify the discrepancies in the statistical significance of associations between adherence types. The study was adequately powered at 86% to detect the average statistically significant Spearman's correlation for overall adherence, but not subtypes of adherence or differences from Mann–Whitney *U* tests. A larger sample would allow for subgroup analysis and control for factors, such as demographics. However, the associations may be nonetheless limited by ceiling effects from the generally high scores on the validated measures used in this study. The sample was also unusual in that it largely comprised of CAM therapists. Interestingly though, they were not necessarily receiving and appraising the same CAM they use to treat patients. The unintended sampling bias occurred due to the lack of forums for CAM patients in the United Kingdom willing to participate in the study, requiring access to the intended population through CAM practitioners. The other limitation is that the survey had not been validated to test the TCM Consultation Model for Adherence and not all novel findings at the lowest level of detail were tested as to not overburden patients with questions. However, validated questionnaires were used where appropriate, and their Cronbach's alphas that were reported in the literature were similar to those in this study.

## 5. Conclusions

The TCM Consultation Model for Adherence was not determined to be applicable for CAM as a whole, but statistically significant associations were found between factors within the model and overall adherence. There was a positive association between patients feeling supported in the management of their health, patients trusting in their practitioner, and patients having a therapeutic relationship with their practitioner and overall adherence. Although there are also differences between the types of adherence, certain behaviours appear to be significant throughout, which is advantageous for designing interventions. Further testing of such behaviours in the TCM Consultation Model for Adherence is recommended for intervention development.

## Figures and Tables

**Table 1:** Participant sociodemographics.

Background characteristic	Number of participants (%) (total: 101)
Age [median (range)]	[49 (21–74)]

Gender	
Male	10 (9.9)
Female	91 (90.1)

Ethnicity	
White	87 (86.1)
Asian	8 (7.9)
Mixed	3 (3.0)
Black	3 (3.0)

Education	
Secondary	6 (5.9)
College	10 (9.9)
Diploma	25 (24.8)
Bachelor	35 (34.7)
Master	20 (19.8)
Doctoral	5 (5.0)

Occupation	
Human health and social work activities	43 (42.6)
Unemployed	17 (16.8)
Multiple	9 (8.9)
Professional, scientific, and technical activities	9 (8.9)
Education	7 (6.9)
Administrative and support service activities	4 (4.0)
Arts, entertainment, and recreation	3 (3.0)
Other service activities	3 (3.0)
Self-employed	3 (3.0)
Construction	1 (1.0)
Wholesale and retail trade; repair of motor vehicles and motorcycles	1 (1.0)
Accommodation and food service activities	1 (1.0)

Income	
Less than £10000	31 (30.7)
£10000–19999	21 (20.8)
£20000–29999	25 (24.8)
£30000–39999	11 (10.9)
£40000–49999	4 (4.0)
£50000–59999	3 (3.0)
£60000 or more	6 (5.9)

**Table 2:** Participant health problems.

Health problem	Number of participants (%) (total: 101)
Diseases of the musculoskeletal system and connective tissue	31 (30.7)
Multiple	16 (15.8)
General health and wellbeing	12 (11.9)
Factors influencing health status and contact with health services	6 (5.9)
Symptoms, signs, and abnormal clinical and laboratory findings, not elsewhere classified	6 (5.9)
Treatment modality	6 (5.9)
Mental, behavioral, and neurodevelopmental disorders	4 (4.0)
Diseases of the nervous system	4 (4.0)
Injury, poisoning, and certain other consequences of external causes	4 (4.0)
Neoplasms	2 (2.0)
Endocrine, nutritional, and metabolic diseases	2 (2.0)
Diseases of the respiratory system	2 (2.0)
Diseases of the digestive system	2 (2.0)
Diseases of the genitourinary system	1 (1.0)
Diseases of the blood and blood-forming organs and certain disorders involving the immune mechanism	1 (1.0)
Diseases of the circulatory system	1 (1.0)
External causes of morbidity	1 (1.0)
Duration of days the therapist was seen [median (range)]	[1095 (1–7300)]

**Table 3:** The most popular CAM treatment modalities that were being used and have been previously tried.

Most popular CAM	Number of participants currently receiving treatment modality from a therapist (%) (total: 101)	Number of participants previously tried treatment modality (%) (total: 101)
Massage	48 (47.5)	52 (51.5)
Reflexology	21 (20.8)	52 (51.5)
Acupuncture	20 (19.8)	51 (50.5)
Yoga	19 (18.8)	52 (51.5)
Aromatherapy	16 (15.8)	39 (38.6)
Meditation	15 (14.9)	50 (49.5)

**Table 4:** The CARE Measure.

The CARE Measure item	Median (IQR) (number of participants: 101)
Making you feel at ease	5 (5–5)
Letting you tell your “story”	5 (5–5)
Really listening	5 (5–5)
Being interested in you as a whole person	5 (5–5)
Fully understanding your concerns	5 (4–5)
Showing care and compassion	5 (5–5)
Being positive	5 (4–5)
Explaining things clearly	5 (4–5)
Helping you to take control	5 (4–5)
Making a plan of action with you	5 (4–5)
Summary score	48 (44–50)

Note: each item is on a scale of 1, which is poor, to 5, which is excellent.

**Table 5:** The Perception of Therapist subscale.

The Perception of Therapist subscale item	Median (IQR) (number of participants: 101)
I trust my therapist	7 (6–7)
I have conﬁdence that my therapist is well qualiﬁed to treat me	7 (6–7)
My therapist is a competent provider of my treatment	7 (6–7)
I am comfortable talking to my therapist about my health problem	7 (7–7)
My therapist wants to help me with my health problem	7 (7–7)
When my therapist talks about my health problem, it does not make sense to me [R]	7 (7–7)
My therapist is an expert in my treatment	6 (5–7)
My therapist is interested when i talk about my health problem	7 (6–7)
My therapist knows how to treat my health problem	6 (6–7)
My therapist provides explanations of my treatment that make sense to me	7 (6–7)
Summary score	67 (61–70)

Note: each item is on a scale of 1 (not at all) to 7 (completely). [R] indicates that the item is reverse scored.

**Table 6:** Additional items.

Item	Median (IQR) (number of participants: 101)
The clinic has a relaxing atmosphere	7 (6–7)
My therapist has a strong reputation	7 (6–7)
My therapist reassures me	7 (6–7)
My therapist shies away from physical contact [R]	7 (5–7)
My therapist has, at times, overstepped what I think are his or her limits [R]	7 (7–7)
I have a good relationship with my therapist	7 (6–7)
My therapist shares the same values as me	6 (5–7)
My therapist tailors my treatment to be most suitable for me	7 (6–7)
My therapist checks my treatment to make sure it is safe and effective	7 (6–7)
My therapist offers advice beyond the immediate health problem	5 (4–7)

Note: each item is on a scale of 1, which is not at all, to 7, which is completely. [R] indicates that the item is reverse scored.

**Table 7:** Therapist traits.

Therapist trait	Number of participants (%) (total: 101)
My therapist is professional	98 (97.0)
My therapist is calm	97 (96.0)
My therapist is a good person	92 (91.1)
My therapist is confident	92 (91.1)
My therapist is of the same gender	59 (58.4)
My therapist is a wise healer	52 (51.2)
My therapist is funny	43 (42.6)
My therapist is of the same culture	41 (40.6)
My therapist is like a friend	38 (37.6)
My therapist is a technician	26 (25.7)

Note: each item is a tick box, which is selected when applicable.

**Table 8:** Statistically significant findings for each type of adherence under aspects of the TCM consultation model for adherence.

Overall adherence	Appointment adherence	Lifestyle advice adherence	Remedy adherence
(Patients feeling comfortable)	(Patients feeling comfortable)		(Patients feeling comfortable)
Opening patients up about themselves	Opening patients up about themselves		Opening patients up about themselves

(Patients feeling valued as individuals)	(Patients feeling valued as individuals)	(Patients feeling valued as individuals)	(Patients feeling valued as individuals)
Tailoring treatment		Tailoring treatment	Tailoring treatment
Checking treatment	Checking treatment	Checking treatment	Checking treatment

(Patients feeling understood)	(Patients feeling understood)	(Patients feeling understood)	
Empathising			
(i) Taking concerns seriously			
Empathising	Empathising	Empathising	
(ii) Touching appropriately	(i) Touching appropriately	(i) Touching appropriately	

(Patients feeling known)	(Patients feeling known)	(Patients feeling known)	(Patients feeling known)
Presenting in a manner suited to the patient	Presenting in a manner suited to the patient	Presenting in a manner suited to the patient	Presenting in a manner suited to the patient
(i) Enthusiastic	(i) Enthusiastic	(i) Enthusiastic	(i) Enthusiastic

Patients feeling supported in the management of their health	Patients feeling supported in the management of their health	Patients feeling supported in the management of their health	Patients feeling supported in the management of their health
Example: making a plan of action (not part of the model)		Example: making a plan of action (not part of the model)	
Educating	Educating	Educating	Educating
(i) Explaining in a way that makes sense to the patient (2 of 3 items)	(i) Explaining in a way that makes sense to the patient (2 of 3 items)	(i) Explaining in a way that makes sense to the patient (2 of 3 items)	(i) Explaining in a way that makes sense to the patient (2 of 3 items)
Helping as much as possible	Helping as much as possible	Helping as much as possible	Helping as much as possible
Helping as much as possible	Helping as much as possible	Helping as much as possible	Helping as much as possible
(ii) Knowing limits	(ii) Knowing limits	(ii) Knowing limits	(ii) Knowing limits
		Helping as much as possible	
		(iii) Adopting role required, for example, wise healer (not part of the model)	

Patients trusting in their practitioner	Patients trusting in their practitioner		Patients trusting in their practitioner
Example trait: well qualified (not part of the model)	Example trait: well qualified (not part of the model)		Example trait: well qualified (not part of the model)
Example trait: competent (not part of the model)	Example trait: competent (not part of the model)		Example trait: competent (not part of the model)
Example trait: expert (not part of the model)	Example trait: expert (not part of the model)		
Example trait: knows how to treat the patient's health problem (not part of the model)	Example trait: knows how to treat the patient's health problem (not part of the model)		
Example trait: of the same culture as the patient	Example trait: of the same culture as the patient		

Patients having a therapeutic relationship with their practitioner		(Patients having a therapeutic relationship with their practitioner)	Patients having a therapeutic relationship with their practitioner
Sharing the same views and values on health		Sharing the same views and values on health	
Perception of therapist (not part of the model)	Perception of therapist (not part of the model)	Perception of therapist (not part of the model)	Perception of therapist (not part of the model)

Note: Brackets containing an aspect of the TCM Consultation Model for Adherence indicate that the finding at the category level is not statistically significant but findings under the category are statistically significant.

**Table 9:** Spearman correlation coefficients and *p* values for each item by the adherence type.

Item	Rho for overall adherence (*p* value) (number of participants: 101)	Rho for appointment adherence (*p* value) (number of participants: 92)	Rho for lifestyle advice adherence (*p* value) (number of participants: 87)	Rho for remedy adherence (*p* value) (number of participants: 49)
Patients feeling cared for “Showing care and compassion”	0.237 (0.017)	0.251 (0.016)	0.164 (0.129)	0.258 (0.074)

Patients feeling comfortable	0.108 (0.281)	0.154 (0.143)	0.102 (0.347)	0.199 (0.171)
“Making you feel at ease”				

Opening patients up about themselves	0.346 (<0.001)^*∗*^	0.296 (0.004)^*∗*^	0.275 (0.010)	0.462 (0.001)^*∗*^
“I am comfortable talking to my therapist about my health problem”				

Setting up a relaxing environment	0.018 (0.857)	−0.060 (0.569)	−0.050 (0.646)	0.237 (0.101)
“The clinic has a relaxing atmosphere”				

Providing warmth physically and emotionally	0.108 (0.281)	0.154 (0.143)	0.102 (0.347)	0.199 (0.171)
“Making you feel at ease”				

Welcoming	0.108 (0.281)	0.154 (0.143)	0.102 (0.347)	0.199 (0.171)
“Making you feel at ease”				

Patients feeling valued as individuals	0.174 (0.082)	0.211 (0.043)	0.164 (0.130)	0.294 (0.040)
“Being interested in you as a whole person”				

Assessing and treating holistically	0.174 (0.082)	0.211 (0.043)	0.164 (0.130)	0.294 (0.040)
“Being interested in you as a whole person”				

Tailoring treatment	0.274 (0.006)^*∗*^	0.268 (0.010)	0.309 (0.004)^*∗*^	0.389 (0.006)^*∗*^
“My therapist tailors my treatment to be most suitable for me”				

Checking treatment	0.364 (<0.001)^*∗*^	0.352 (0.001)^*∗*^	0.285 (0.007)^*∗*^	0.388 (0.006)^*∗*^
“My therapist checks my treatment to make sure it is safe and effective”				

Patients feeling understood	0.191 (0.056)	0.214 (0.040)	0.225 (0.037)	0.203 (0.161)
“Fully understanding your concerns”				

Communicating well	0.053 (0.598)	0.153 (0.146)	0.070 (0.519)	0.091 (0.533)
(i) Listening				
“Really listening”				

Empathising	0.251 (0.011)	0.198 (0.059)	0.251 (0.019)	0.279 (0.052)
CARE Measure summary score				

Empathising	0.326 (0.001)^*∗*^	0.230 (0.028)	0.268 (0.012)	0.243 (0.092)
(i) Taking concerns seriously				
“My therapist is interested when I talk about my health problem”				

Empathising	0.332 (0.001)^*∗*^	0.280 (0.007)^*∗*^	0.400 (<0.001)^*∗*^	0.161 (0.269)
(i) Touching appropriately				
“My therapist shies away from physical contact”				

Patients feeling known	0.174 (0.082)	0.211 (0.043)	0.164 (0.130)	0.294 (0.040)
“Being interested in you as a whole person”				

Presenting in a manner suited to the patient	1038.000 (0.138) (number of participants in each group: 43 versus 58)	998.000 (0.704) (number of participants in each group: 40 versus 52)	695.000 (0.038) (number of participants in each group: 37 versus 50)	313.500 (0.672) (number of participants in each group: 21 versus 28)
(i) Funny				
“My therapist is funny” (Mann–Whitney *U* (*p* value))				

Presenting in a manner suited to the patient	286.500 (0.117) (number of participants in each group: 92 versus 9)	261.000 (0.232) (number of participants in each group: 84 versus 8)	161.500 (0.017) (number of participants in each group: 79 versus 8)	69.000 (0.469) (number of participants in each group: 45 versus 4)
(i) Confident				
“My therapist is confident” (Mann–Whitney *U* (*p* value))				

Presenting in a manner suited to the patient	102.000 (0.113) (number of participants in each group: 97 versus 4)	101.000 (0.159) (number of participants in each group: 88 versus 4)	86.500 (0.109 (number of participants in each group: 83 versus 4)	44.500 (0.900) (number of participants in each group: 47 versus 2)
(i) Calm				
“My therapist is calm” (Mann–Whitney *U* (*p* value))				

Presenting in a manner suited to the patient	0.403 (<0.001)^*∗*^	0.291 (0.005)^*∗*^	0.321 (0.002)^*∗*^	0.372 (0.009)^*∗*^
(i) Enthusiastic				
“Being positive”				

Patients feeling supported in the management of their health	0.357 (<0.001)^*∗*^	0.352 (0.001)^*∗*^	0.299 (0.005)^*∗*^	0.437 (0.002)^*∗*^
“My therapist wants to help me with my health problem”				
Example: making a plan of action (not part of the model)	0.332 (0.001)^*∗*^	0.226 (0.031)	0.283 (0.008)^*∗*^	0.226 (0.118)
“Making a plan of action with you”				

Reassuring	0.232 (0.020)	0.232 (0.026)	0.215 (0.045)	0.285 (0.047)
“My therapist reassures me”				

Educating
(i) Explaining in a way that makes sense to the patient (3 items)	0.144 (0.149)	0.050 (0.634)	0.126 (0.247)	0.381 (0.007)^*∗*^
“Explaining things clearly”	0.426 (<0.001)^*∗*^	0.422 (<0.001)^*∗*^	0.367 (<0.001)^*∗*^	0.386 (0.006)^*∗*^
“When my therapist talks about my health problem, it does not make sense to me” (reverse item)	0.462 (<0.001)^*∗*^	0.393 (<0.001)^*∗*^	0.420 (<0.001)^*∗*^	0.352 (0.013)
“My therapist provides explanations of my treatment that make sense to me”	0.093 (0.354)	−0.001 (0.990)	0.168 (0.119)	−0.009 (0.951)

Educating	—	—	—	—
(i) Giving a lot of multilevel advice, self-help, or homework				
“My therapist offers advice beyond the immediate health problem”				

Helping as much as possible	0.357 (<0.001)^*∗*^	0.352 (0.001)^*∗*^	0.299 (0.005)^*∗*^	0.437 (0.002)^*∗*^
“My therapist wants to help me with my health problem”				

Helping as much as possible	0.340 (<0.001)^*∗*^	0.351 (0.001)^*∗*^	0.337 (0.001)^*∗*^	0.369 (0.009)^*∗*^
(i) Knowing limits				
“My therapist has, at times, overstepped what I think are his or her limits”				

Helping as much as possible	0.071 (0.478)	0.182 (0.082)	0.049 (0.650)	0.157 (0.282)
(i) Giving time				
“Letting you tell your “story”				

Helping as much as possible	1009.000 (0.785) (number of participants in each group: 26 versus 75)	832.500 (0.686) (number of participants in each group: 23 versus 69)	681.000 (0.454) (number of participants in each group: 24 versus 63)	195.500 (0.694) (number of participants in each group: 9 versus 40)
(i) Adopting role required, for example, technician (not part of the model)				
“My therapist is a technician”(Mann–Whitney *U* (*p*value))				

Helping as much as possible	913.500 (0.011) (number of participants in each group: 52 versus 49)	875.500 (0.102) (number of participants in each group: 47 versus 45)	580.500 (0.002)^*∗*^ (number of participants in each group: 49 versus 38)	239.000 (0.310) (number of participants in each group: 30 versus 19)
(i) Adopting the role required, for example, a wise healer (not part of the model)				
“My therapist is a wise healer” (Mann–Whitney *U* (*p* value))				

Helping as much as possible	853.500 (0.013) (number of participants in each group: 38 versus 63)	853.000 (0.115) (number of participants in each group: 38 versus 54)	687.000 (0.050) (number of participants in each group: 34 versus 53)	259.000 (0.378) (number of participants in each group: 24 versus 25)
(i) Adopting the role required, for example, like a friend				
“My therapist is like a friend”(Mann–Whitney *U* (*p* value))				

Helping as much as possible	32.500 (0.015) (number of participants in each group: 98 versus 3)	30.500 (0.017) (number of participants in each group: 89 versus 3)	66.500 (0.176) (number of participants in each group: 84 versus 3)	9.000 (0.408) (number of participants in each group: 48 versus 1)
(i) Adopting the role required, for example, a professional (not part of the model)				
“My therapist is a professional” (Mann–Whitney *U* (*p* value))				

Imparting responsibility for health	0.255 (0.010)	0.156 (0.137)	0.219 (0.041)	0.319 (0.025)
“Helping you to take control”				

Patients trusting in their practitioner	0.353 (<0.001)^*∗*^	0.329 (0.001)^*∗*^	0.275 (0.010)	0.469 (0.001)^*∗*^
“I trust my therapist”				

Example trait: well qualified (not part of the model)	0.341 (<0.001)^*∗*^	0.324 (0.002)^*∗*^	0.218 (0.042)	0.479 (<0.001)^*∗*^
“I have conﬁdence that my therapist is well qualiﬁed to treat me”				

Example trait: competent (not part of the model)	0.372 (<0.001)^*∗*^	0.346 (0.001)^*∗*^	0.255 (0.017)	0.436 (0.002)^*∗*^
“My therapist is a competent provider of my treatment”				

Example trait: expert (not part of the model)	0.314 (0.001)^*∗*^	0.272 (0.009)^*∗*^	0.185 (0.087)	0.265 (0.066)
“My therapist is an expert in my treatment”				

Example trait: knows how to treat the patient's health problem (not part of the model)	0.333 (0.001)^*∗*^	0.286 (0.006)^*∗*^	0.264 (0.014)	0.237 (0.101)
“My therapist knows how to treat my health problem”				

Example trait: strong reputation	0.121 (0.229)	0.113 (0.284)	0.183 (0.090)	0.124 (0.397)
“My therapist has a strong reputation”				

Example trait: a good person	283.000 (0.107) (number of participants in each group: 92 versus 9)	239.000 (0.322) (number of participants in each group: 85 versus 7)	243.000 (0.543) (number of participants in each group: 80 versus 7)	89.500 (0.510) (number of participants in each group: 44 versus 5)
“My therapist is a good person” (Mann–Whitney *U* (*p* value))				

Example trait: of the same culture as the patient	852.500 (0.007)^*∗*^ (number of participants in each group: 41 versus 60)	707.500 (0.005)^*∗*^ (number of participants in each group: 37 versus 55)	672.500 (0.031) (number of participants in each group: 35 versus 52)	274.000 (0.964) (number of participants in each group: 17 versus 32)
“My therapist is of the same culture” (Mann–Whitney *U* (*p* value))				

Example trait: of the same gender as the patient	1209.000 (0.831) (number of participants in each group: 59 versus 42)	933.000 (0.361) (number of participants in each group: 53 versus 39)	775.000 (0.160) (number of participants in each group: 49 versus 38)	369.000 (0.132) (number of participants in each group: 26 versus 23)
“My therapist is of the same gender”(Mann–Whitney *U* (*p* value))				

Patients having a therapeutic relationship with their practitioner	0.309 (0.002)^*∗*^	0.216 (0.039)	0.226 (0.036)	0.377 (0.008)^*∗*^
“I have a good relationship with my therapist”				

Sharing the same views and values on health	0.282 (0.004)^*∗*^	0.260 (0.012)	0.333 (0.002)^*∗*^	0.232 (0.109)
“My therapist shares the same values as me“				

Perception of therapist	0.483 (<0.001)^*∗*^	0.434 (<0.001)^*∗*^	0.401 (<0.001)^*∗*^	0.371 (0.009)^*∗*^
Summary score (not part of the model)				

^*∗*^indicates a statistically significant association at *p* < 0.01.

## Data Availability

The datasets generated and analysed during the current study are not publicly available due to consent not being given for this purpose by participants.

## Supplementary Materials

Supplementary Materials Appendix A lists all the CAM treatment modalities that were being used and have been previously tried by participants. Appendix B details the associations between aspects of the TCM Consultation Model for Adherence and types of adherence.
