# Supplementary material for: Testing the Traditional Chinese Medicine Consultation Model for Adherence in Complementary and Alternative Medicine
Source: Evid Based Complement Alternat Med. 2020 Dec 30;2020:8897628. doi: 10.1155/2020/8897628 (PMC7790569; doi:10.1155/2020/8897628)
Supplement: Supplementary Materials — Appendix A lists all the CAM treatment modalities that were being used and have been previously tried by participants. Appendix B details the associations between aspects of the TCM Consultation Model for Adherence and types of adherence. [file 8897628.f1.pdf]

Appendix A

Supplementary material on the CAM used and tried by participants

CAM	Number of participants currently receiving treatment modality from therapist (%) (total: 101)	Number of participants previously tried treatment modality (%) (total: 101)
Amatsu	0 (0.0)	1 (1.0)
Acupuncture	20 (19.8)	51 (50.5)
Acupressure	10 (9.9)	21 (20.8)
Alexander technique	0 (0.0)	12 (11.9)
Aromatherapy	16 (15.8)	39 (38.6)
Art therapy	3 (3.0)	1 (1.0)
Autogenic training	0 (0.0)	0 (0.0)
Ayurveda	3 (3.0)	14 (13.9)
Bach flower remedies	5 (5.0)	34 (33.7)
Biochemic tissue salts	4 (4.0)	8 (7.9)
Bioresonance	1 (1.0)	0 (0.0)
Biorhythms	0 (0.0)	1 (1.0)
Bowen therapy	3 (3.0)	3 (3.0)
Breathing techniques	0 (0.0)	1 (1.0)
Cannabis remedies	1 (1.0)	0 (0.0)
Chiropractic	9 (8.9)	31 (30.7)
Chelation and cell therapy	0 (0.0)	0 (0.0)
Colonic irrigation	1 (1.0)	8 (7.9)
Colour therapy	2 (2.0)	8 (7.9)
CoMra therapy	0 (0.0)	1 (1.0)
Craniosacral therapy	4 (4.0)	2 (2.0)
Crystal and gem therapy	4 (4.0)	21 (20.8)
Cupping	1 (1.0)	1 (1.0)
Dance movement therapy	0 (0.0)	5 (5.0)
Dry needling	1 (1.0)	0 (0.0)
Eden energy medicine	1 (1.0)	1 (1.0)
Emmett technique	1 (1.0)	2 (2.0)
Esoteric connective tissue therapy	1 (1.0)	0 (0.0)
Flower essence therapy	0 (0.0)	1 (1.0)
Healing	9 (8.9)	27 (26.7)
Herbal medicine	10 (9.9)	30 (29.7)
Homeopathy	7 (6.9)	39 (38.6)
Hypnosis	6 (5.9)	25 (24.8)
Kinesiology	0 (0.0)	4 (4.0)
Magnetic therapy	2 (2.0)	7 (6.9)
Massage	48 (47.5)	52 (51.5)
Meditation	15 (14.9)	50 (49.5)
Muscle activation technique	0 (0.0)	1 (1.0)
Music therapy	2 (2.0)	4 (4.0)
Myofascial release	4 (4.0)	2 (2.0)
Naturopathy	4 (4.0)	9 (8.9)
Nutritional therapy	10 (9.9)	15 (14.9)
Osteopathy	9 (8.9)	38 (37.6)
Ozone therapy	1 (1.0)	1 (1.0)
Pilates	0 (0.0)	1 (1.0)
Qi gong	0 (0.0)	1 (1.0)
Rebirthing	0 (0.0)	1 (1.0)
Reiki	9 (8.9)	41 (40.6)
Reflexology	21 (20.8)	52 (51.5)
Relaxation	11 (10.9)	26 (25.7)
Rolfing	0 (0.0)	1 (1.0)
Seichem	0 (0.0)	1 (1.0)
Shamanism	0 (0.0)	1 (1.0)
Shiatsu	4 (4.0)	26 (25.7)
Sophrology	0 (0.0)	1 (1.0)
Soul journey therapy	0 (0.0)	1 (1.0)
Spiritual healing	2 (2.0)	19 (18.8)
Tai ji	0 (0.0)	1 (1.0)
Talk therapies and counselling	11 (10.9)	41 (40.6)
Traditional Chinese medicine	8 (7.9)	25 (24.8)
Therapeutic touch	5 (5.0)	3 (3.0)
Visualization	4 (4.0)	13 (12.9)
Voice and sound therapy	3 (3.0)	8 (7.9)
Yoga	19 (18.8)	52 (51.5)
Zero balancing	0 (0.0)	1 (1.0)

Appendix B

Supplementary material on hypotheses testing

For each hypothesis, the relationship between aspects of the TCM Consultation Model for Adherence and adherence to CAM was investigated:

1. *There is no association between patients feeling cared for and overall adherence.*

   Patients feeling cared for was not associated with adherence (for overall adherence $r_s = 0.237$, $p = 0.017$; for appointment adherence $r_s = 0.251$, $p = 0.016$; for lifestyle advice adherence $r_s = 0.164$, $p = 0.129$; for remedy adherence $r_s = 0.258$, $p = 0.074$).

2. *There is no association between patients feeling comfortable and overall adherence.*

   Even though patients feeling comfortable was also not associated with adherence (for overall adherence $r_s = 0.108$, $p = 0.281$; for appointment adherence $r_s = 0.154$, $p = 0.143$; for lifestyle advice adherence $r_s = 0.102$, $p = 0.347$; for remedy adherence $r_s = 0.199$, $p = 0.171$), one of the example behaviours showed weak to moderate correlation across all types of adherence except for lifestyle advice. This was opening patients up about themselves (for overall adherence $r_s = 0.346$, $p < 0.001$; for appointment adherence $r_s = 0.296$, $p = 0.004$; for remedy adherence $r_s = 0.462$, $p = 0.001$).

3. *There is no association between patients feeling valued as individuals and overall adherence.*

   Patients feeling valued as individuals was not associated with adherence either (for overall adherence $r_s = 0.174$, $p = 0.082$; for appointment adherence $r_s = 0.211$, $p = 0.043$; for lifestyle advice adherence $r_s = 0.164$, $p = 0.130$; for remedy adherence $r_s = 0.294$, $p = 0.040$). However, two of three example behaviours showed weak correlation across all types of adherence except for appointments. Checking treatment was correlated with all types of adherence (for overall adherence $r_s = 0.364$, $p < 0.001$; for appointment adherence $r_s = 0.352$, $p = 0.001$; for lifestyle advice adherence $r_s = 0.285$, $p = 0.007$; for remedy adherence $r_s = 0.388$, $p = 0.006$), while tailoring treatment was correlated with adherence types aside from appointments (for overall adherence $r_s = 0.274$, $p = 0.006$; for lifestyle advice adherence $r_s = 0.309$, $p = 0.004$; for remedy adherence $r_s = 0.389$, $p = 0.006$).

4. *There is no association between patients feeling understood and overall adherence.*

   Patients feeling understood was not associated with adherence as well (for overall adherence $r_s = 0.191$, $p = 0.056$; for appointment adherence $r_s = 0.214$, $p = 0.040$; for lifestyle advice adherence $r_s = 0.225$, $p = 0.037$; for remedy adherence $r_s = 0.203$, $p = 0.161$). One of the tested behaviours that support the feeling was statistically significant overall, but not for individual types of adherence. Taking concerns seriously was the behaviour only weakly correlated with overall adherence ($r_s = 0.326$, $p = 0.001$). Touching appropriately was weakly to moderately correlated with adherence overall ($r_s = 0.332$, $p = 0.001$) but also specifically for appointments ($r_s = 0.280$, $p = 0.007$) and lifestyle advice ($r_s = 0.400$, $p < 0.001$). No aspect of feeling understood was shown to be correlated with remedy adherence.

5. *There is no association between patients feeling known and overall adherence.*

   Patients feeling known was not associated with adherence too (for overall adherence $r_s = 0.174$, $p = 0.082$; for appointment adherence $r_s = 0.211$, $p = 0.043$; for lifestyle advice adherence $r_s = 0.164$, $p = 0.130$; for remedy adherence $r_s = 0.294$, $p = 0.040$). However, enthusiasm or being positive was weakly to moderately correlated with adherence (for overall adherence $r_s = 0.403$, $p < 0.001$; for appointment adherence $r_s = 0.291$, $p = 0.005$; for lifestyle advice adherence $r_s = 0.321$, $p = 0.002$; for remedy adherence $r_s = 0.372$, $p = 0.009$). This example behaviour that enables patients to feel known was statistically significant across all types of adherence.

6. *There is a positive association between patients feeling supported in the management of their health and overall adherence.*

   Patients feeling supported in the management of their health was associated with adherence (for overall adherence $r_s = 0.357$, $p < 0.001$; for appointment adherence $r_s = 0.352$, $p = 0.001$; for lifestyle advice adherence $r_s = 0.299$, $p = 0.005$; for remedy adherence $r_s = 0.437$, $p = 0.002$). The correlation was weak to moderate across the types of adherence. Helping as much as possible (representative of patients feeling supported in the management of their health), but knowing limits were two of the items tested under this factor that showed statistically significant association across all types of adherence (for overall adherence $r_s = 0.340$, $p < 0.001$; for appointment adherence $r_s = 0.351$, $p = 0.001$; for lifestyle advice adherence $r_s = 0.337$, $p = 0.001$; for remedy adherence $r_s = 0.369$, $p = 0.009$). The correlations were weak, except for remedy adherence and helping as much as possible, where it was moderate. The way patients' therapists talk about health problems made sense to patients was another item that was weakly to moderately correlated with adherence (for overall adherence $r_s = 0.426$, $p < 0.001$; for appointment adherence $r_s = 0.422$, $p < 0.001$; for lifestyle advice adherence $r_s = 0.367$, $p < 0.001$; for remedy adherence $r_s = 0.386$, $p = 0.006$). However, the other two items that test for the behaviour of explaining in a way that makes sense to the patient differed in their statistical significance. Providing explanations of treatment that make sense was moderately correlated with all types of adherence except remedy (for overall adherence $r_s = 0.462$, $p < 0.001$; for appointment adherence $r_s = 0.393$, $p < 0.001$; for lifestyle advice adherence $r_s = 0.420$, $p < 0.001$). Yet, only remedy adherence was correlated with explaining things clearly, albeit weakly ($r_s = 0.381$, $p = 0.007$).

   Although not part of the TCM Consultation Model, making a plan of action was a behaviour that enabled patients to feel supported in the management of their health and was weakly correlated with overall adherence ($r_s = 0.332$, $p = 0.001$) as well as for lifestyle advice specifically ($r_s = 0.283$, $p = 0.008$). Similarly, patients who thought their therapist was a wise healer adhered to lifestyle advice differently to those who did not perceive their therapist to adopt this role (Mann-Whitney $U = 580.500$, $p = 0.002$). Other items relating to patients feeling supported in the management of their health did not reveal statistically significant differences.

7. *There is a positive association between patients trusting in their practitioner and overall adherence.*

   Patients trusting in their practitioner was associated with adherence overall ($r_s = 0.353$, $p < 0.001$). The correlation was weak overall and for appointment adherence ($r_s = 0.329$, $p = 0.001$), but moderate for remedy adherence ($r_s = 0.469$, $p = 0.001$).

Patients who were of the same culture as their therapist overall adhered differently to those who had therapists of a different culture (Mann-Whitney U = 852.500, p = 0.007). This was seen specifically with appointment adherence as well (Mann-Whitney U = 707.500, p = 0.005).

Although not from the TCM Consultation Model, four example traits that enabled patients to trust their practitioner were weakly correlated with adherence overall and specifically to appointments. These were being well-qualified to treat the patient (for overall adherence $r_s = 0.341$, $p < 0.001$; for appointment adherence $r_s = 0.324$, $p = 0.002$), a competent provider of the patient's treatment (for overall adherence $r_s = 0.372$, $p < 0.001$; for appointment adherence $r_s = 0.346$, $p = 0.001$) and an expert in the patient's treatment (for overall adherence $r_s = 0.314$, $p = 0.001$; for appointment adherence $r_s = 0.272$, $p = 0.009$) as well as knowing how to treat the patient's health problem (for overall adherence $r_s = 0.333$, $p = 0.001$; for appointment adherence $r_s = 0.286$, $p = 0.006$). Additionally, being well-qualified to treat the patient and a competent provider of the patient's treatment were moderately correlated with remedy adherence (for well-qualified to treat $r_s = 0.479$, $p < 0.001$; for competent provider of treatment $r_s = 0.436$, $p = 0.002$).

8. *There is a positive association between patients having a therapeutic relationship with their practitioner and overall adherence.*

   Patients having a therapeutic relationship with their practitioner was associated with adherence overall ($r_s = 0.309$, $p = 0.002$). The correlation was also weak in remedy adherence ($r_s = 0.377$, $p = 0.008$). However, sharing the same views and values on health, which can help build the therapeutic relationship, was only weakly correlated with overall adherence ($r_s = 0.282$, $p = 0.004$) and lifestyle advice ($r_s = 0.333$, $p = 0.002$).

Where there was a statistically significant association, the correlation was always positive.
